# Supplementary material for: Mapping the evolution of fertility support policies in China: A content and instrumental analysis
Source: PLoS One. 2025 Oct 9;20(10):e0332137. doi: 10.1371/journal.pone.0332137 (PMC12510515; doi:10.1371/journal.pone.0332137)
Supplement: S1 Appendix — (ZIP) [file pone.0332137.s001.zip › S1 Appendix. 226 original policy documents/1-国家计生委、国家经贸委、公安部、民政部、国家工商行政管理局等部门关于进一步做好城市计划生育工作的意见(FBM-CLI-4-26864).doc]

国家计生委、国家经贸委、公安部、民政部、国家工商行政管理局等部门关于进一步做好城市计划生育工作的意见

**发布部门：** [国家计划生育委员会(已更名)](https://www.pkulaw.com/law/chl?Aggs.IssueDepartment=60314) [国家经济贸易委员会(已变更)](https://www.pkulaw.com/law/chl?Aggs.IssueDepartment=60313) [公安部](https://www.pkulaw.com/law/chl?Aggs.IssueDepartment=60205) [民政部](https://www.pkulaw.com/law/chl?Aggs.IssueDepartment=60208) [国家工商行政管理总局(已撤销)](https://www.pkulaw.com/law/chl?Aggs.IssueDepartment=60402) [机构沿革](javascript:void(0))

**发文字号：**国计生政[1997]第26号

**发布日期：**1997.03.11

**实施日期：**1997.03.11

**时效性：** [现行有效](https://www.pkulaw.com/law/chl?Aggs.TimelinessDic=01)

**效力级别：** [部门规范性文件](https://www.pkulaw.com/law/chl?Aggs.EffectivenessDic=XE0302)

**法规类别：** [计划生育管理](https://www.pkulaw.com/law/chl?Aggs.Category=09705)

国家计生委、国家经贸委、公安部、民政部、人事部、
劳动部、卫生部、国家工商行政管理局关于
进一步做好城市计划生育工作的意见
（国计生政[1997]第26号）

各省、自治区、直辖市、计划单列市计生委、经贸委（经委、计经委）、公安厅（局）、民政厅（局）、人事厅（局）、劳动厅（局）、卫生厅（局）、工商局：
　　伴随着经济发展和社会进步，我国计划生育工作取得了举世瞩目的成就，城市（主要指城区，下同）人口已基本实现了向低出生、低死亡、低自然增长的人口再生产类型的转变。城市居民的婚育观念发生了显著变化，计划生育已经成为大多数群众的自觉行为。卓有成效的城市计划生育工作不仅减轻了因人口增长过快对经济和社会发展的压力，促进了人口素质和居民生活质量的提高，而且为全国控制人口过快增长，平抑人口出生高峰做出了巨大贡献，对农村计划生育工作起到了示范、带动和辐射作用。随着改革开放的不断深化和社会主义市场经济体制的逐步建立，我国城市计划生育工作也面临着许多新情况和新问题。巩固与发展城市计划生育工作的成果，对于完成到本世纪末和到２０１０年控制人口的目标，促进两个文明建设具有重要意义。为此，提出以下意见。
　　一、进一步做好城市计划生育工作是一项长期任务
　　当前，城市计划生育工作面临着许多新情况、新问题。城市数量增多，规模扩大，流动人口大量增加；不少企业在转换经营机制的过程中，撤并或削减了计划生育机构和人员；许多企业事业单位无行政主管部门；在市场竞争中出现了一大批下岗职工；在城市开发建设、旧城改造的过程中一大批居民处于人户分离状态，这都使计划生育工作的难度增加。街道办事处和居民委员会担负的计划生育工作任务越来越重，但机构、编制及干部队伍素质等都难以适应工作的需要。同时，随着生活水平和文化素质不断提高，城市居民对避孕节育、优生优育、生殖保健的需求也越来越高。而目前城市计划生育的宣传教育、技术服务以及管理方法还存在着一些薄弱环节和亟待解决的问题，不能满足人们的需求。还要看到，目前城市的低生育水平在一定程度上是依靠行政制约措施实现的。如果放松计划生育工作或者对某些问题处理不当，妇女生育水平仍有可能出现不正常的回升。因此，必须高度重视城市计划生育工作面临的新情况、新问题，充分认识进一步做好城市计划生育工作仍然是一项长期的任务。

　　二、城市计划生育工作的指导思想和目标
　　城市计划生育工作正处在一个重要的改革与发展时期。今后一个时期，城市计划生育工作的指导思想和目标是：以邓小平建设有中国特色社会主义理论为指导，坚持人口与经济、社会协调发展和可持续发展的战略，坚持全心全意为人民服务的宗旨，在全国率先实现计划生育工作思路、工作方法的“两个转变”，改革、完善管理体制，加强、充实基层基础工作，努力深化宣传教育，逐步扩大服务领域，不断提高管理水平和服务质量，使城市计划生育工作与改革开放和社会主义市场经济体制相适应，与广大育龄群众不断增长的需求相适应，以期稳定城市人口的低生育水平，提高人口质量，改善人口结构，促进人的全面发展和家庭的文明幸福，为城市的社会主义现代化建设创造良好的人口环境。
　　要从各地实际出发，通过改革，逐步建立适应社会主义市场经济体制的计划生育管理体制，形成单位负责、各部门参与，以市、区和街道为主管理的新局面；逐步完善能满足育龄群众需求的计划生育服务体系，形成依托社区，各相关部门、群众团体优势互补、密切协作的新局面；改进计划生育管理方法，逐步建立更加科学、合理的管理制度。在大中城市的城区，逐步做到符合条例规定的一孩生育不再申报审批，而由基层主动纳入人口出生计划；在加强宣传教育和指导的基础上，积极推广知情选择避孕措施；根据城市计划生育工作的特点，改进和简化人口计划管理程序和评估考核方法。改革要积极稳妥地进行，要通过试点取得经验以后再逐步推开。要坚持分类指导，对不同规模、不同类型、不同工作基础的城市，应提出不同的要求。

　　三、改革和完善城市计划生育工作管理体制
　　总结多年来的实践经验，城市计划生育工作要实行“单位负责，条条保证，以块为主，条块配合”的管理体制。
　　“单位负责”就是国家机关、企业事业单位、社会团体等都要依据[宪法](https://www.pkulaw.com/chl/208e8dcf5c64cbc8bdfb.html)和有关法律、法规，认真贯彻落实计划生育基本国策，担负起做好计划生育工作的责任。这是做好城市计划生育工作的基础。“条条保证”就是要求各部门对本系统所辖单位的计划生育工作提出明确的目标和要求，提供必要的条件，督促所属单位贯彻落实计划生育基本国策。这是多年来城市计划生育工作行之有效的经验。“以块为主”就是指市、区政府及其派出机关街道办事处，要按照行政区划对本辖区所有单位和居民委员会的计划生育工作切实负起领导的责任，主要承担下达任务、检查监督和协调、服务等职能。这是城市计划生育工作管理体制发展的必然趋势。“条块配合”就是指要处理好“条”、“块”之间的关系，“条”与“块”要在明确任务、各负其责的前提下，加强相互协调与配合。这是做好城市计划生育工作必不可少的条件。城市所有的“单位”、“条条”、“块块”都要主动地、认真地履行各自的职责，齐心协力把计划生育工作做好。

　　四、进一步加强城市基层计划生育工作网络建设
　　街道办事处负责管理本辖区内单位和居民委员会的计划生育工作，是做好城市基层计划生育工作的重要环节。街道办事处要实行人口与计划生育目标管理责任制。要建立健全本辖区人口与计划生育统计信息系统，做好计划生育管理与服务，落实计划生育政策；宣传党和国家的计划生育政策、法律、法规，开展人口与计划生育基础知识教育；负责本辖区避孕药具的管理和发放，组织辖区内的单位和居民委员会为育龄夫妇落实避孕节育措施；负责出具、查验本地流出和外地流入人员的计划生育证明，做好流动人口的计划生育工作；组织协调有关部门在社区开展与计划生育有关的服务工作。街道办事处应设立计划生育工作机构并配备必要的工作人员。
　　居民委员会是居民自我管理、自我教育、自我服务的基层群众性自治组织，有责任协助人民政府或者街道办事处做好计划生育工作。居民委员会主要负责做好本居住地区不属于机关、团体、部队、企业事业单位的育龄群众的计划生育工作；与企业事业单位共同做好下岗人员的计划生育工作；与原居住地区共同做好暂住本居住地区人户分离人员的计划生育工作。要逐步改变目前居民委员会干部人数偏少、年龄偏大、待遇偏低，以及一些居民委员会无人管理计划生育工作的状况。居民委员会应有专人负责计划生育工作；并保证必需的经费投入。
　　市、区政府各部门要指导本系统所辖单位根据实际情况，建设好基层计划生育工作网络。各单位都要有必要的人员负责计划生育工作。
　　要充分发挥工会、共青团、妇联等群众团体在城市基层计划生育工作中的作用，特别要重视和发挥计划生育协会组织群众自我教育、自我管理和自我服务的作用。要在集贸市场和流动人口集中的地方，在“三资”、私营和个体企业中建立计划生育协会，充分发挥其作用。

　　五、认真做好城市计划生育宣传教育和技术服务工作
　　计划生育是社会主义精神文明建设的重要组成部分。要根据党中央关于社会主义精神文明建设总的指导思想，把计划生育纳入社会主义精神文明建设的总体规划和部署之中，同各地广泛开展的各项群众性精神文明创建活动紧密结合起来，并作为创建文明家庭、文明单位、文明社区、文明城市的一项重要内容和重要考核指标。
　　要大力加强城市计划生育的宣传教育，认真贯彻落实中宣部、[国家计生委等１３个部门１９９６年联合发出的《关于进一步加强计划生育宣传教育工作的通知](https://www.pkulaw.com/chl/c2f54da93ac81a6bbdfb.html)》，组织协调宣传、教育、广播电视、新闻出版、文化艺术以及科技、卫生等有关部门，制定城市计划生育宣传教育规划，充分利用社会各方面的力量和大众传媒开展计划生育宣传教育。要根据城市大众传媒和文化设施比较多、群众文化教育水平比较高的特点和人民群众不断增长的需求，进一步普及人口与计划生育基础知识教育，广泛开展以实现人口与经济、社会协调发展和可持续发展为主要内容的国情国策教育；以倡导晚婚晚育、少生优生、男女平等、生男生女都一样、计划生育丈夫有责和优育优教为主要内容的家庭美德教育；以提高育龄群众自我保健意识和自我保健能力，维护育龄妇女合法权益为主要内容的生殖健康教育；以保护环境、预防疾病、降低出生缺陷发生率、提高出生人口素质为主要内容的生命科学教育。要不断提高宣传教育的质量，讲求针对性、实效性。要把面对大众的宣传教育同面对个人的咨询服务结合起来。要重视向未婚青年进行性道德和性健康教育并提供必要的咨询服务。城市中学要开展人口教育和青春期教育。各级党校、干校要开设人口理论课。要办好各类人口学校、婚育学校。要在城市中形成广泛的计划生育宣传教育网络，满足不同人群多层次、多方面的需求。
　　要大力加强城市计划生育技术服务工作。大中城市城区的计划生育技术服务工作主要依靠医疗卫生机构。社区卫生服务网络要把做好避孕节育、优生优育、生殖保健的宣传、咨询和规定的技术服务当作自己的一项重要职责，努力提高辖区内育龄夫妇的避孕率，提高技术服务质量，降低人工流产率和节育手术并发证发症发生率。要改进工作方法和工作作风，提高服务质量，主动关心和了解育龄群众的避孕节育情况，结合技术服务搞好宣传、咨询，帮助育龄夫妇落实避孕节育措施。卫生部门要协助计划生育部门加强对城市计划生育技术服务工作的管理。在贯彻实施《[母婴保健法](https://www.pkulaw.com/chl/ea633acd04298a40bdfb.html)》中努力做好提高出生人口素质的工作。各级医院和妇幼保健院（所）都要设立计划生育科（室）或计划生育门诊，配备合格的技术力量和必要的设备，确保提供优质服务。
　　城市计划生育部门的服务机构着重做好避孕节育、优生优育、生殖保健的宣传、咨询和指导、提供有关人口与计划生育的知识和信息等综合服务工作，并在医疗卫生机构服务覆盖不到或比较薄弱的地区和人群中开展节育技术服务。要不断提高技术水平和服务质量。在力所能及的范围内，可以适当拓宽服务领域，依法围绕生育、节育、不育开展有关的妇幼保健工作。具体服务项目由县（区）级以上地方政府征求卫生部门和计划生育部门的意见后确定。大中城市视自身的条件和需要，可依托医疗卫生机构或计划生育科研所、技术服务机构设立计划生育技术服务指导中心和协调小组，承担技术指导、新技术推广、专业技术人员培训等工作。
　　计划生育部门要加强对计划生育技术服务工作的总体规划、组织协调和监督检查，充分发挥各方面卫生技术力量的作用，共同做好计划生育技术服务工作。要协调有关部门制定计划生育技术服务的工作职责、服务规范和考核办法，按国家有关政策规定，妥善解决从事计划生育技术服务的专业人员的职称、待遇等问题。要加强城市避孕药具的管理和供应，保证供应渠道畅通以满足群众的需求。进一步开发、完善、规范避孕药具市场。计划生育部门和卫生部门要建立联席会议制度，经常交流信息，通报情况，共同研究解决计划生育技术服务工作中的问题。

　　六、努力做好企业的计划生育工作
　　企业（包括国有、集体、私营、“三资”企业和其他企业）的计划生育工作是城市计划生育工作的重要组成部分。各类企业都要根据[宪法](https://www.pkulaw.com/chl/208e8dcf5c64cbc8bdfb.html)和有关法律、法规的规定，认真贯彻落实计划生育基本国策，担负起做好计划生育工作的责任。要把计划生育工作纳入企业管理之中，作为企业精神文明建设的一个重要方面，把计划生育工作同关心职工身心健康和家庭的文明幸福结合起来。企业党委一把手和企业法定代表人或行政领导要对计划生育工作亲自抓、负总责，并建立计划生育工作责任制。要根据本企业计划生育工作的需要，建立切实可行的工作制度，保证计划生育各项工作落到实处。
　　企业要做好各类下岗人员的计划生育宣传教育，向他们提出计划生育要求；加强与下岗人员所居住的街道、居民委员会的联系，及时通报情况，与街道、居民委员会密切配合，关心下岗人员的生产、生活、生育，帮助他们排忧解难，共同做好计划生育工作。

　　七、切实做好流动人口的计划生育工作
　　城市的各级政府要把流动人口的计划生育工作纳入对流动人口实行综合治理的总体方案之中，纳入人口与计划生育目标管理责任制之中。要根据中央办公厅和国务院办公厅《关于转发〈[中央社会治安综合治理委员会关于加强流动人口管理工作的意见〉的通知](https://www.pkulaw.com/chl/d992207ee21ea528bdfb.html)》（厅字〔１９９５〕４２号）精神，组织协调各有关部门实行统一领导，统一管理，统一收费，对流动人口的计划生育工作实行综合治理。公安、劳动、工商行政管理等部门在审批办理、查验有关证件时，要核查经现居住地计划生育部门查验过的流动人口计划生育证明。对没有计划生育证明或没有查验证明的，公安部门在按规定办理暂住登记、发放暂住证的同时，应要求其补办并通报当地计划生育部门；劳动部门暂不办理流动就业证；工商行政管理部门对申请从事个体经营的，暂不核发营业执照。卫生、交通、建设、民政等有关部门都要配合计划生育部门共同做好流动人口计划生育工作。各有关部门要建立协查、通报制度，或采取联合办公的形式，及时沟通情况，协调行动。
　　流动人口的计划生育工作由现居住地和户口所在地政策，各负其责，相互配合，共同管理。现居住地对流动人口计划生育工作的日常管理和服务负主要责任，要负责查验流动人口计划生育证明，掌握育龄人员的计划生育情况，帮助流动人口育龄夫妇落实避孕节育措施，做好经常性的管理工作。要增强为流动人口服务的意识，积极为流动人口提供良好的避孕节育和生殖保健服务，尽可能为他们排忧解难，注意保障他们的合法权益。户口所在地要对流动人口进行人口与计划生育宣传教育，帮助和指导流动人口中的育龄夫妇落实避孕节育措施，如实出具流动人口计划生育证明，努力提高发证率。流动人口现居住地和户口所在地要加强联系。要加强对流动人口计划生育工作的考核，既要考核流动人口现居住地，又要考核户口所在地。
　　计划生育部门要认真履行职责，担负起做好流动人口计划生育工作的重任，要做好党委、政府的参谋、助手，协助政府组织协调各有关部门各负其责，互相配合，共同做好流动人口计划生育的管理和服务工作。

　　八、坚持和完善城市人口与计划生育目标管理责任制
　　各级党委、政府要高度重视城市计划生育工作，坚持党政一把手亲自抓、负总责，坚持实行人口与计划生育目标管理责任制。要逐步完善人口与计划生育目标管理指标体系，使其切合实际、科学合理。城市计划生育工作的评估考核指标，要从以考核对人口数量的控制为主逐步转到以考核计划生育服务质量为主。要在稳定低生育水平的前提下，把考核重点放在提高工作水平和服务质量上，把广大育龄群众对计划生育工作的满意程度，作为评价工作效果的一项重要指标。考核办法要简便务实，坚决反对形式主义和弄虚作假。对一个单位、一个地区计划生育工作的考核要采取平时检查和年终考核相结合的方法，做到评估客观、准确、公正，并兑现奖惩。要加快建立计算机管理系统的步伐，运用现代信息技术推进计划生育工作的科学管理。

　　九、建设高素质的计划生育干部队伍
　　各级党委、政府要切实加强计划生育干部队伍建设。要选派政治业务素质好、年富力强的干部到计划生育部门担任领导职务，把各级计划生育领导班子建设成为政治坚定、勤政廉洁、勇于改革、求真务实、结构合理、团结协作的领导集体；要选派懂政策、会管理、有文化、有能力、热心计划生育工作的同志到街道、居民委员会从事计划生育工作，努力建设一支思想好、作风正、懂业务、会管理的计划生育干部队伍。要采取切实措施，稳定计划生育干部队伍。
　　要加强计划生育系统干部职工的政治思想教育和业务学习，坚持用马克思列宁主义、毛泽东思想和邓小平建设有中国特色社会主义理论武装全体干部职工，深入持久地开展爱国主义和艰苦创业精神的教育，大力加强全心全意为人民服务的教育，还要不断拓宽知识面，掌握做好工作所需要的各种知识和技能。各地要做出规划，在几年内使现有干部普遍轮训一次，还要有计划地进行学历、岗位培训和专业技术培训。
　　要加强职业道德建设，大力倡导敬业爱岗、忠于职守、爱护群众、服务群众、脚踏实地、求真务实、尊重科学、钻研业务、遵纪守法、廉洁奉公的职业道德。要结合实际情况，采取多种方式，对广大干部职工经常深入地进行职业责任、职业道德、职业纪律的教育，切实加强思想政治工作，引导大家树立正确的世界观、人生观、价值观，培育优良的职业道德，坚决纠正行业不正之风，规范职业行为，树立行业新风。
　　　　　　　　　　　　　　　　　　　　　　　　　　一九九七年三月十一日

© 北大法宝：（[www.pkulaw.com](https://www.pkulaw.com/)) 专业提供法律信息、法学知识和法律软件领域各类解决方案。北大法宝为您提供丰富的参考资料，正式引用法规条文时请与标准文本核对。
欢迎查看所有[产品和服务](https://www.pkulaw.com/helps/2.html)。法宝快讯：[如何快速找到您需要的检索结果？](https://www.pkulaw.com/helps/70.html)[法宝](https://www.pkulaw.com/helps/69.html)V6有何新特色？

扫描二维码阅读原文

**原文链接：**<https://www.pkulaw.com/chl/de712ec95b2191a9bdfb.html>
